# Phase 1 Single and Multiple Dose Clinical Trials (ASCENT‐1 and ASCENT‐2) for Dose‐Finding of PRX012 in Participants with Biologically Confirmed Alzheimer’s Disease

**DOI:** 10.1002/alz.090487

**Published:** 2025-01-09

**Authors:** Chad Swanson, Ferenc Martényi, Ryan E. Tooker, Brian M. Campbell, Ann Johnson, Lingnan Li, Mary E. Quiceno, Wagner M. Zago, Gene G. Kinney, Hideki Garren

**Affiliations:** ^1^ Prothena Biosciences Inc., Brisbane, CA USA

## Abstract

**Background:**

Alzheimer’s disease (AD) is a progressive neurodegenerative disorder marked by the presence of brain amyloid beta (Aβ) plaques and stages of memory loss, cognitive decline, psychological and psychiatric changes, inability to perform activities of daily living, dementia, and eventually death. Recent evidence demonstrates the slowing of clinical decline with plaque‐clearing, anti‐Aβ monoclonal antibodies. PRX012 is a humanized monoclonal antibody that targets and clears known pathogenic forms of Aβ in development for subcutaneous (SC) use. Ongoing phase 1 clinical trials are evaluating the optimal dose of PRX012 in participants with AD.

**Method:**

PRX012‐101 (ASCENT‐1) is a phase 1, randomized, double‐blind, placebo‐controlled, single ascending dose clinical trial to evaluate the safety, tolerability, immunogenicity, and pharmacokinetics of PRX012 in healthy volunteers and participants with biologically confirmed AD (via amyloid PET). Subjects are given a single SC injection of PRX012, from 70 mg up to 400 mg, or placebo and followed for 85 days.

PRX012‐102 (ASCENT‐2) is a phase 1, randomized, double‐blind, sponsor‐open, placebo‐controlled, multiple dose clinical trial to evaluate the safety, tolerability, immunogenicity, pharmacokinetics, and pharmacodynamics of PRX012 in participants with biologically confirmed AD. Participants are assigned to one of two groups based on APOE genotype: Group A subjects with AD who are heterozygous‐ or non‐carriers of APOE4 alleles, and Group B subjects with AD who are homozygous for APOE4. Cohorts of APOE4 homozygous subjects were incorporated to allow for better representation and more complete characterization of the profile of PRX012 in homozygous carriers, as these individuals make up a small proportion of typical Alzheimer’s study populations. Subjects are given a series of 6 monthly SC injections of PRX012, from 45 mg up to 400 mg, or placebo.

**Result:**

Data from both ASCENT‐1 and ASCENT‐2 will support dose‐finding for PRX012 through generation of a robust dataset.

**Conclusion:**

Phase 1 development of PRX012 will support future late‐stage clinical trials that have the potential to position PRX012 as a best‐in‐class, next‐generation once‐monthly subcutaneous antibody for the treatment of AD.

